# Bladder Cancer: New Insights into Its Molecular Pathology

**DOI:** 10.3390/cancers10040100

**Published:** 2018-04-01

**Authors:** Kentaro Inamura

**Affiliations:** Division of Pathology, The Cancer Institute, Japanese Foundation for Cancer Research, 3-8-31 Ariake, Koto-ku, Tokyo 135-8550, Japan; kentaro.inamura@jfcr.or.jp; Tel.: +81-3-3570-0111 (ext. 5604); Fax: +81-3-3570-0558

**Keywords:** APOBEC, FGFR3, GATA3, immune checkpoint inhibitor, molecular pathological epidemiology, PD-L1, precision medicine, The Cancer Genome Atlas (TCGA), uroplakin, urothelial carcinoma

## Abstract

Bladder cancer is one of the most prevalent cancers worldwide. Unfortunately, there have been few advances in its clinical management due to a poor understanding of the correlations between its molecular and clinical features. Mounting evidence suggests that bladder cancer comprises a group of molecularly heterogeneous diseases that undergo a variety of clinical courses and possess diverse therapeutic responses. Owing to the close association between its molecular subtypes and clinicopathological features, specific therapeutic strategies have recently been suggested. This review summarizes the current understanding of the molecular pathology of bladder cancer, including its molecular biomarkers/pathways and molecular subtypes that have been newly identified using high-throughput technologies. It also discusses advances in our understanding of personalized treatments for specific molecular subtypes.

## 1. Introduction

**Figure 1:**
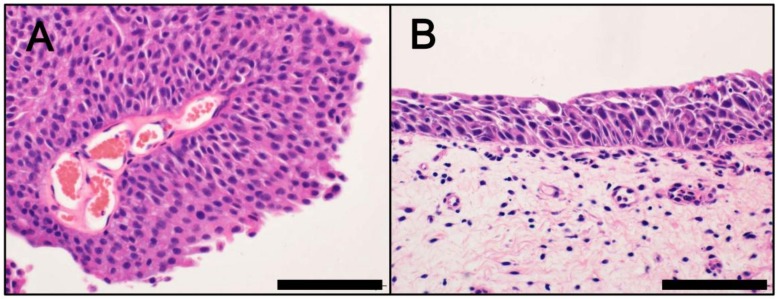
Morphology of non-invasive bladder cancer (hematoxylin and eosin staining). (**A**) Non-invasive papillary tumor (pTa); (**B**) Carcinoma in situ (CIS; pTis). Scale bar = 100 µm.

Bladder cancer is one of the most prevalent cancers globally, with approximately 400,000 new cases diagnosed each year [1]. Incidence rates are 3‒4 times higher in men than in women. Patients are typically at an age of 65–70 years at the time of diagnosis. The main risk factor for bladder cancer is environmental or occupational exposure to carcinogens, with smoking identified as the most likely causative agent [1,2]. Approximately 70–80% patients with newly diagnosed bladder cancer present with a form of non-muscle-invasive bladder cancer (NMIBC), such as non-invasive papillary tumor (pTa) (Figure 1A), carcinoma in situ (CIS; pTis) (Figure 1B), or early invasive tumor (non-muscle invasive; pT1). These tumors characteristically recur in 50–70% of cases, with only approximately 10–20% of cases progressing to muscle-invasive bladder cancer (MIBC) [3,4,5]. Accumulating evidence suggests that bladder cancer represents a group of molecularly and clinicopathologically heterogeneous diseases [6,7,8,9,10,11,12,13,14,15,16,17,18,19,20,21,22,23]. An increased understanding of the molecular pathology of bladder cancer has led to the identification of specific molecular subtypes. This review provides a summary of the current understanding of the molecular pathology of bladder cancer. It also discusses potential therapies for specific molecular subtypes.

## 2. Molecular Biomarkers and Pathways

The molecular biomarkers and pathways involved in bladder cancer are key to understanding its biological heterogeneity and identifying specific subtypes that can be used to predict clinical outcomes and treatment responsiveness to personalized therapies.

### 2.1. Mutation Spectrum

Bladder cancer commonly harbors chromosomal aberrations, which are characterized by aneusomies, deletions and amplifications that affect almost all chromosomes [24,25]. The deletion of chromosome nine is prevalent not only in bladder cancer but also in urothelial hyperplasia and dysplasia, suggesting that this deletion occurs during the earliest stages of bladder tumorigenesis [26,27].

TCGA data demonstrated that MIBC shows high overall mutation rates (mean 8.2 and median 5.8 per megabase), which are similar to those of melanomas and non-small cell lung cancers; most mutations appear to be passenger mutations without any functional consequences [13]. The high number of somatic mutations is dominated by a C:G→T:A transition in the context of TpC dinucleotides, which is characteristic of mutations caused by the APOBEC family of cytidine deaminases [28], which typically represses the propagation of viruses [6]. Recurrent genetic alterations include mutations in the *TP53*, *FGFR3*, *PIK3CA* and *RB1* genes [13]. Bladder cancer also frequently harbors somatic *TERT* promoter mutations, which occur early in the process of bladder carcinogenesis [5,29,30]. Given that telomere shortening acts as a mitotic clock, the activation of telomerase, which elongates telomeres at the ends of chromosomes, is crucial for the continued growth of cancer cells [31]. The Cancer Genome Atlas (TCGA) cohort study of MIBC demonstrated the mutual exclusiveness of alterations between the *CDKN2A* and *TP53*, *CDKN2A* and *RB1*, *TP53* and *MDM2* and *FGFR3* and *RB1* gene pairs. Similar analyses showed the co-occurrence of mutations in the *TP53* and *RB1* genes and in the *FGFR3* and *CDKN2A* genes [13].

TCGA data have provided the comprehensive molecular characterization of MIBCs [13]; however, the generated results need to be carefully evaluated. TCGA data revealed 58 significantly mutated genes and high frequencies of occurrence of several genetic pathways; however, given the heterogenic nature of MIBCs, the examination of even larger samples is essential to obtain a complete catalogue of mutations or pathways, including those with low frequencies [32]. Creating a reasonably comprehensive catalogue of candidate cancer genes mutated in ≥2% of patients with MIBCs will require thousands of samples, given the high overall mutation rates of these cancers [33]. Considering the reduced costs of sequencing, a more comprehensive catalogue of MIBCs is likely to be generated in the near future [32]. Further, almost no studies have replicated the results of the TCGA study [13]; therefore, it is important that the results be confirmed by other cohorts. Moreover, because the TCGA study [13] examined chemotherapy-naïve samples, the generated results provide limited insight into tumor evolution and the mechanisms of acquired therapeutic resistance, which remain to be elucidated [34]. Further studies are required to construct a more comprehensive and precise molecular catalogue of MIBCs for developing therapeutic strategies.

### 2.2. FGFR3/RAS Pathway

The FGFR3/RAS pathway can undergo activation in bladder tumors at any stage but is predominantly active in low-grade NMIBC. Up to 80% of NMIBCs harbor activating point mutations in *FGFR3*, which has been associated with both a higher risk of recurrence in non-invasive papillary bladder cancer and favorable clinical outcomes in pT1 tumors [5,6,35,36,37]. *FGFR3* mutations have been identified in only 10–20% of MIBC cases. Mutant *FGFR3* activates the RAS-MAPK pathway, leading to cell proliferation. Approximately 10% of bladder cancer cases harbor mutations in *RAS* genes, such as *HRAS*, *KRAS* and *NRAS* [38], which do not occur with *FGFR3* mutations [39]. Emerging evidence has shown that a certain subset of bladder cancers harbor recurrent in-frame *FGFR3*-*TACC3* fusions [12,40].

### 2.3. PIK3/AKT/MTOR Pathway

The PIK3/AKT/MTOR pathway regulates important steps in tumorigenesis and tumor progression. This pathway is activated by receptor tyrosine kinases including ERBB2, ERBB3 and FGFR3. *PIK3CA* mutations, which have been identified in 22% of MIBC cases, are more commonly located in the helical domain than in kinase domain, likely due to the mutagenic activity of APOBEC. *PIK3CA* mutations also appear to be associated with favorable outcomes in patients who undergo radical cystectomy [41]. The upstream pathway activator *ERBB2* is amplified, mutated, or overexpressed in a subset of MIBC cases [2,12]. *ERBB2* mutations (12% of MIBC cases) are commonly found in the extracellular domain and are also likely due to the APOBEC mutational signature [13]. The deletion or reduced expression of PTEN, which is a negative regulator of the PIK3/AKT/MTOR pathway, has been observed in many MIBC cases, whereas *AKT1* and *TSC1*, both of which also regulate this pathway, are not as frequently mutated [5].

### 2.4. TP53/RB1 Pathway

The TP53/RB1 pathway plays an important role in the regulation of cell cycle progression. The mutation or deletion of *TP53* has been observed predominantly in cases of CIS and MIBC [5,26,42]. *RB1* inactivation has also been observed predominantly in MIBC, often with concurrent *TP53* mutations [7].

According to TCGA cohort data [13], 89% of MIBCs have an inactivated TP53 cell cycle pathway, with *TP53* mutations in 48%, *MDM2* amplification in 6% and *MDM2* overexpression in 19% of cases. On the other hand, 17% of MIBC cases harbor *RB1* mutations. *CDKN2A* (*p16*), which functions as a negative regulator of the RB1 pathway, was found to be mutated (7%) or deleted (22%).

## 3. Divergent Pathways

**Figure 2:**
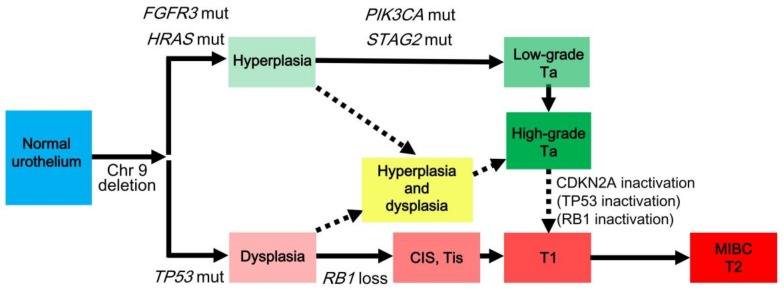
Potential pathways of the tumorigenesis and tumor progression of bladder cancer [2,6,25]. Bladder cancer develops either via divergent pathways comprising either the FGFR3/RAS pathway (green) or the TP53/RB1 pathway (red). Chromosome nine deletion occurs in the early phase of tumorigenesis. *FGFR3*/*HRAS* mutation frequently occurs during the development of hyperplasia. In case of low-grade Ta carcinoma with recurrent *PIK3CA*/*STAG2* mutation, hyperplasia develops into high-grade Ta carcinoma, which may progress to become T1 carcinoma after CDKN2A inactivation or TP53/RB1 inactivation. *TP53* mutation frequently occurs during the development of dysplasia. Dysplasia may develop into CIS (Tis) after *RB1*, followed by T1 carcinoma. T1 carcinoma progresses to become MIBC (T2) after various genomic alterations. Chr, chromosome; CIS, carcinoma in situ; MIBC, muscle-invasive bladder cancer; mut, mutation.

Bladder cancer is believed to develop via a field effect that involves multiple sites in the bladder mucosa, leading to multifocal and metachronous tumorigenesis [5,43,44]. Bladder epithelial cells in the affected field become malignant by developing genetic alterations that lead to carcinogenesis by clonal evolution [45]. Bladder cancer develops either via the FGFR3/RAS pathway or the TP53/RB1 pathway [5,25] (Figure 2). The deletion of chromosome 9 is prevalent in urothelial hyperplasia and dysplasia [26,27,46], suggesting that this deletion occurs in both the pathways. The FGFR3/RAS pathway enables tumors to progress from urothelial hyperplasia to non-invasive papillary tumors with high recurrence rates. The *FGFR3*/*HRAS* mutation frequently occurs during the development of urothelial hyperplasia [2,6,25,30,47,48]. Low-grade Ta carcinoma frequently harbors the *PIK3CA*/*STAG2* mutation [2,6,25,49] and develops into a high-grade Ta carcinoma, which may progress to become T1 carcinoma after CDKN2A inactivation [2,6,13,25,50]. The TP53/RB1 pathway allows tumors to progress from dysplasia to invasive tumors via CIS [25]. The *TP53* mutation frequently occurs during the development of urothelial dysplasia [26]. *RB1* loss appears to allow the progression from urothelial hyperplasia to CIS (Tis) [2,6,25,51]. Lesion with hyperplasia and dysplasia (hyperplastic lesion with cytological atypia) may play a role in interactions between both the pathways as shown in Figure 2 [2,5,6,25].

## 4. Classification by Molecular Subtype

Bladder cancer is molecularly and clinicopathologically heterogeneous. Its heterogeneity hinders the development of personalized medicine in the form of molecularly targeted therapies. Genome-wide expression and molecular profiling studies have been performed to categorize bladder cancer into intrinsic subgroups that are associated with specific molecular features, prognoses and responses to certain therapies [7,8,9,10,11,12,13,14].

### 4.1. NMIBC

A comprehensive transcriptional analysis of 460 NMIBC cases demonstrated that these tumors can be categorized into three genomic subtypes (classes 1–3) that significantly differ with respect to their clinicopathological features, including the rate of progression-free survival [14]. These 460 cases, which consist of 345 pTa, 112 pT1 and 3 CIS tumors, together with 16 MIBC cases, were examined. Class 1 (*n* = 96) and class 2 (*n* = 235) tumors demonstrated a luminal gene expression signature, which included the expression of uroplakins. Class 1 tumors mainly included non-invasive papillary tumors and resulted in the best prognosis among the three different classes. Class 1 tumors had the highest expression levels of early cell cycle genes. Class 2 tumors contained a higher number of pT1 and high-grade cases and had the worst prognosis among the three classes. In fact, class 2 tumors were found to share a gene expression signature with the majority of concurrently examined MIBCs. They also had high expression levels of *KRT20*, which are substantially associated with CIS lesions [52]. Additionally, class 2 tumors were characterized by epithelial-mesenchymal transition (EMT)-associated, stem cell-associated and APOBEC mutational signatures and were found to contain mutations in *TP53* and *ERCC2*. In NMBC, the presence of an APOBEC-related mutational signature was associated with a poor prognosis. Class 3 tumors (*n* = 129) expressed a basal-like gene expression signature, which includes the expression of *KRT5*, *KRT15* and *CD44* [2,4,14].

### 4.2. MIBC

**Figure 3:**
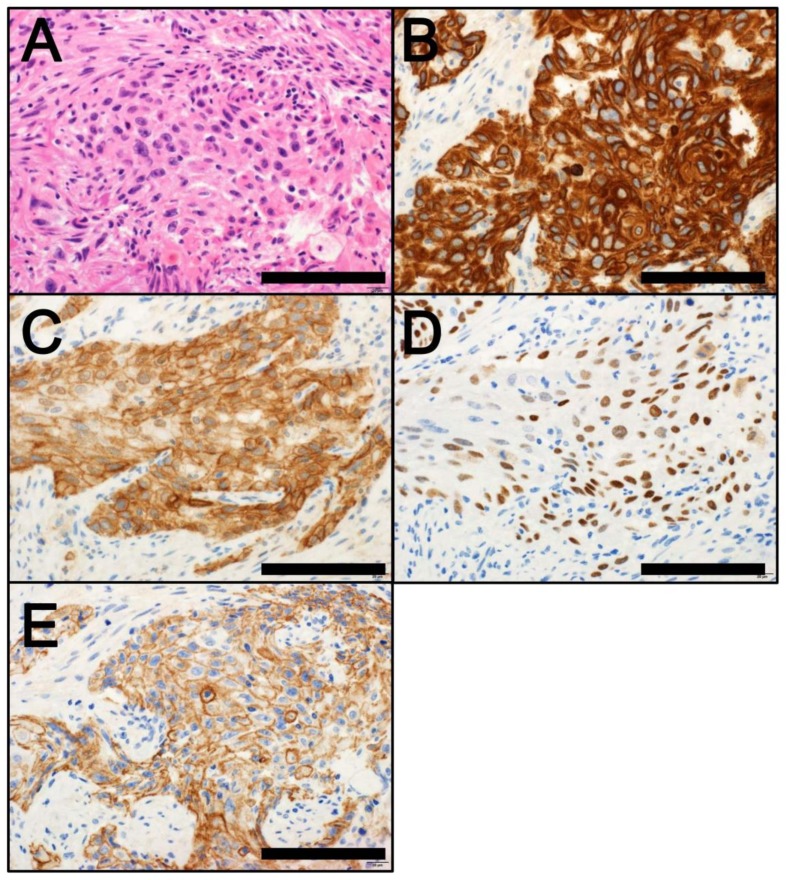
(**A**) Basal type of bladder cancer (hematoxylin and eosin staining); (**B**) CK5/6 immunostaining (cytoplasmic; positive); (**C**) CD44 immunostaining (membranous; positive); (**D**) TP63 immunostaining (nuclear; positive); (**E**) EGFR immunostaining (membranous; positive). Scale bar = 100 µm.

**Figure 4:**
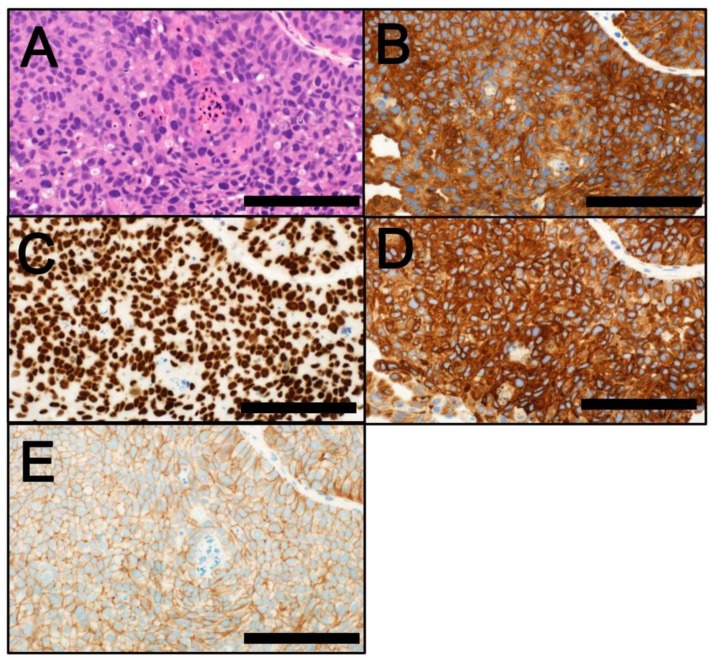
(**A**) Luminal type of bladder cancer (hematoxylin and eosin staining); (**B**) KRT20 immunostaining (cytoplasmic; positive); (**C**) GATA3 immunostaining (nuclear; positive); (**D**) Uroplakin II immunostaining (cytoplasmic and membranous; positive); (**E**) ERBB2 immunostaining (membranous; positive). Scale bar = 100 µm.

The comprehensive molecular characterization of MIBCs has allowed their categorization into subtypes that are associated with specific clinicopathological features [6,7,8,9,10,11,12,13]. Bladder cancer has been categorized into two major subtypes—luminal and basal—that share a similarity with intrinsic types of breast cancer [53]. Molecular profiling studies have further classified bladder cancer by the risk of recurrence and progression, as well as by treatment response [7,8,9,10,11,12,13]. Lindgren et al. [10,11] first identified a keratinized/basal type of MIBC that was associated with a poor clinical outcome. Choi et al. [9] demonstrated that while the basal type of MIBC (Figure 3A) is aggressive, it responds to neoadjuvant chemotherapy (NAC). It is also characterized by the expression of CK5/6 (Figure 3B), CD44 (Figure 3C), TP63 (Figure 3D) and EGFR (Figure 3E) proteins but not of KRT20 or markers of urothelial differentiation. These authors also demonstrated that the luminal type of MIBC (Figure 4A) is characterized by *FGFR3* mutations and the protein expression of KRT20 (Figure 4B) and markers of urothelial differentiation (e.g., GATA3 (Figure 4C), uroplakins (Figure 4D) and ERBB2 (Figure 4E)) but not of CK5/6, CD44, TP63, or EGFR proteins. They also identified a TP53-like MIBC subtype that is consistently resistant to NAC [9]. In 2014, TCGA data from 131 MBIC cases was used to categorize bladder cancer into four expression subtypes (clusters I‒IV). Clusters I and II share features of the luminal type of MIBC, including urothelial cell differentiation and expression of GATA3 and FOXA1. High expression levels of E-cadherin (CDH1) and *miR-200* family members (which inhibit EMT) [54] are also found in clusters I and II. Cluster I (a papillary-like subtype) is characterized by a papillary morphology, *FGFR3* alterations and low expression levels of *miR-99a-5p* and *miR-100-5p*, which downregulate *FGFR3* expression. Cluster III (a basal/squamous-like subtype) shows features of the basal type of MIBC, including squamous cell differentiation and enrichment of stem cell expression features. Class IV, corresponding to an EMT subtype, expresses low levels of E-cadherin and members of the *miR-200* family. In terms of treatment response, the TCGA cluster II/TP53-like luminal subtype is highly sensitive to the PD-L1 (CD274) inhibitor atezolizumab but not to NAC [9,12,55]. Patients with TCGA cluster II/TP53-like tumors can thus be spared from unnecessary NAC, instead undergoing cystectomy or immunotherapy without delay [9,12].

In 2017, TCGA data from 412 MIBC cases permitted the comprehensive cataloging of their molecular and clinicopathological features [13]. Using whole-exome sequencing, five mutational signatures were identified. Two were variants of the APOBEC mutational signature and accounted for 67% of all single-nucleotide variants. Furthermore, levels the APOBEC mutational signature were associated with the expression levels of *APOBEC3A* and *APOBEC3B* as previously reported [56]. Most identified bladder cancer mutations were clonal, suggesting that the APOBEC mutational signature occurs early in carcinogenesis. Elucidation of the mechanism by which this signature affects tumor progression may help prevent the incidence of such cancers [13]. Cluster analyses identified four clusters of mutational signatures, which were associated with the rate of overall survival. Patients with a cluster with high APOBEC mutational signature and high mutational burden had a five-year survival rate of 75% compared with the cluster with the lowest mutational burden (five-year survival rate of 22%). The exceptionally high survival rate of the former cluster may be the result of more efficacious antitumor immune reactions of the host to tumors with high mutational burdens.

**Figure 5:**
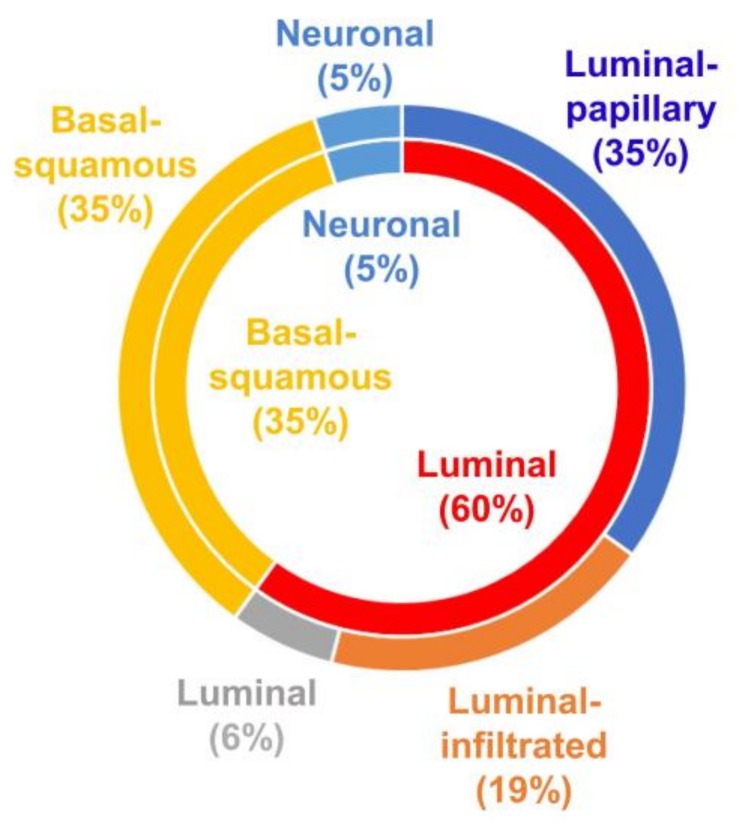
The mRNA-based subtypes of muscle-invasive bladder cancer (MIBC) by The Cancer Genome Atlas consortium [13]. MIBC can be divided into five subtypes [luminal-papillary (35%), luminal-infiltrated (19%), luminal (6%), basal-squamous (35%) and neuronal (5%)].

**Figure 6:**
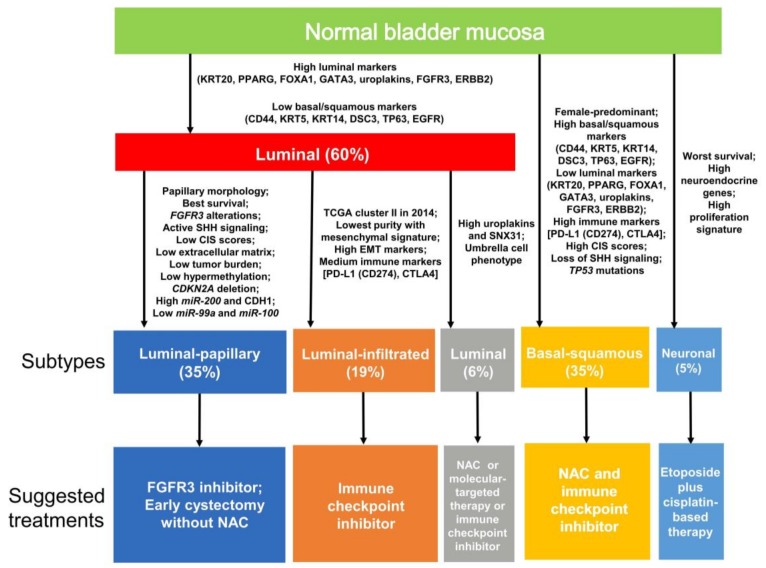
Categorization of muscle-invasive bladder cancer into five different subtypes based on mRNA expression by The Cancer Genome Atlas consortium [13]. Molecular and clinicopathological characteristics and suggested treatments for the five subtypes are summarized. CIS, carcinoma in situ; EMT, epithelial-mesenchymal transition; NAC, neoadjuvant chemotherapy; SHH, sonic hedgehog.

Clustering of mRNA expression levels allowed the identification of five molecularly distinct MIBC subtypes (i.e., luminal-papillary, luminal-infiltrated, luminal, basal-squamous and neuronal) (Figure 5) that can be used to stratify patients according to the predicted biological behavior and predicted treatment response of the tumor (Figure 6). For example, the luminal-papillary, luminal-infiltrated and luminal subtypes all express luminal markers, including *GATA3*, *FOXA1*, uroplakins and *KRT20*. 

The luminal-papillary subtype (35%) is characterized by a papillary morphology and results in the best overall survival. It characteristically possesses *FGFR3* alterations, including *FGFR3-TRCC3* fusions and active sonic hedgehog (SHH) signaling. This subtype frequently has a low CIS score, low levels of tumor-associated extracellular matrix and smooth muscle, a low mutational burden, low levels of hypermethylation and a high frequency of *CDKN2A* deletions. These tumors express high levels of *miR-200* family members, CDH1 and ERBB2 but low levels of *miR-99a-5p* and *miR-100-5p* [32].

The luminal-infiltrated subtype (19%) has the lowest tumor purity and a mesenchymal expression signature. These tumors highly express EMT markers and moderately express immune markers (*PD-L1* and *CTLA4*).

The luminal subtype (6%) expresses high levels of *uroplakins* (*UPK1A* and *UPK2*), *KRT20* and *SNX31* and displays an umbrella cell phenotype.

The basal-squamous subtype (35%) corresponds to the previously defined basal subtype, which is associated with squamous cell differentiation and basal keratin expression. This subtype is predominately found in females and expresses high levels of basal markers (*CD44*, *KRT5*, *KRT6A* and *KRT14*), squamous cell differentiation markers (*TGM1*, *DSC3* and *PI3*) and immune markers (*PD-L1* and *CTLA4*). This subtype frequently has a high CIS score, loss of SHH signaling and presence of *TP53* mutations.

The neuronal subtype (5%) lacks a neuroendocrine morphology in most cases and results in the worst clinical outcomes of all the subtypes. This subtype expresses high levels of neuroendocrine and neuronal genes, possesses a high proliferation signature and has a large number of *TP53* and *RB1* mutations.

## 5. Molecular Alterations and Their Therapeutic Relevance

Few therapeutic advances have been made for MIBC over the past two decades and clinical outcomes have not changed significantly. Cytotoxic cisplatin-based chemotherapy continues to be the first-line treatment for advanced or metastatic bladder cancer. Bladder cancer comprises a group of molecularly heterogeneous diseases that undergo a variety of clinical courses and possess diverse therapeutic responses [57]. Although increased survival can be observed in 5–10% of patients in response to NAC [58,59], it remains impossible to predict patient response to NAC. In an era of precision medicine, molecular subtype-specific therapeutic strategies are needed.

In addition to the previous molecular subtypes of bladder cancer [7,8,9,10,11,12,41], TCGA data from 412 MIBC cases have been used to explore the likely success of available therapies (Figure 6) [13,34]. The luminal-papillary subtype is predicted to have little response to NAC. For this subtype, FGFR inhibitors or early cystectomy without NAC is suggested [9,60]. The luminal-infiltrated subtype is also predicted to have little response to NAC. According to a previous clinical study [55], TCGA cluster II [12], which corresponds to the luminal-infiltrated subtype, elicits a substantial response to immune checkpoint therapy with atezolizumab; thus, immune checkpoint inhibitors are suggested. There are no recommended therapies at present for the novel luminal subtype. Studies of its sensitivity to NAC, molecularly targeted therapy and immune checkpoint inhibitors are ongoing. The basal-squamous subtype has a relatively high sensitivity to NAC. Both NAC and immune checkpoint inhibitors may be appropriate therapeutic options for this subtype. For the neuronal subtype, etoposide plus cisplatin-based therapy is suggested, similarly to treatments for neuroendocrine cancers at other sites. These therapeutic suggestions inform clinical trial designs and hold the potential to advance the treatment of bladder cancer. Validation of the suggested therapeutic strategies by clinical trials is required.

Immunotherapy has emerged as a promising strategy for the treatment of various malignancies, including bladder cancer [34,55,61,62,63,64]. Mounting evidence indicates that immune checkpoint mechanisms play a critical role in the suppression of the anti-tumor T-cell-mediated immune response. The phase III clinical trial KEYNOTE-045 demonstrated that the PD-1 (PDCD1) antibody pembrolizumab was associated with a significantly longer overall survival with a lower rate of treatment-associated adverse events than chemotherapy as a second-line therapy for platinum-refractory advanced urothelial carcinoma [62]. In this trial, the efficacy of pembrolizumab appeared to be independent of PD-L1 expression in tumor cells and infiltrating immune cells. Furthermore, the PD-1 antibody pembrolizumab (KEYNOTE-052 [63]) and the PD-L1 antibody atezolizumab (IMvigor 210 [64]) demonstrated encouraging durable anticancer activity in a subset of cisplatin-ineligible chemotherapy-naïve patients with metastatic urothelial cancer [34]. The clinical trials of immune checkpoint inhibitors have yielded promising results. An elucidation of the association between the benefit from immune checkpoint inhibitors and the newly-identified molecular subtypes of MIBCs is anticipated.

## 6. Conclusions and Future Directions

This review summarizes the current knowledge of the molecular pathology of bladder cancer, including molecular biomarkers/pathways and newly identified molecular subtypes. Advances in the treatment of bladder cancer are lacking compared to those in other malignancies. Recent advances in our understanding of the molecular characteristics of bladder cancers will potentially help in their evolution from a poorly understood, heterogeneous group of diseases with variable clinical courses and therapeutic responses toward more specific and molecularly-characterized subtypes. Moreover, the identification of molecularly-defined subtypes may enable the implementation of tailored therapies and better patient management. The identified associations of specific molecular subtypes with therapeutic strategies are not yet validated but can inform the design of current clinical trials. The translation of the molecular characterization of bladder cancer to clinical applications is required to provide clinicians with the best therapeutic options for patients with bladder cancer.

## Abbreviations

CIS	carcinoma in situ
EMT	epithelial-mesenchymal transition
MIBC	muscle-invasive bladder cancer
NAC	neoadjuvant chemotherapy
NMIBC	non-muscle-invasive bladder cancer
SHH	sonic hedgehog
TCGA	The Cancer Genome Atlas
